# Preconception Dietary Inflammatory Index and Risk of Gestational Diabetes Mellitus Based on Maternal Body Mass Index: Findings from a Japanese Birth Cohort Study

**DOI:** 10.3390/nu14194100

**Published:** 2022-10-02

**Authors:** Hyo Kyozuka, Tsuyoshi Murata, Hirotaka Isogami, Karin Imaizumi, Toma Fukuda, Akiko Yamaguchi, Shun Yasuda, Akiko Sato, Yuka Ogata, Mitsuaki Hosoya, Seiji Yasumura, Koichi Hashimoto, Hidekazu Nishigori, Keiya Fujimori, the Japan Environment and Children’s Study (JECS) Group

**Affiliations:** 1 Department of Obstetrics and Gynecology, School of Medicine, Fukushima Medical University, 1 Hikarigaoka, Fukushima 960-1295, Japan; 2 Fukushima Regional Center for the Japan Environmental and Children’s Study, 1 Hikarigaoka, Fukushima 960-1295, Japan; 3 Department of Pediatrics, School of Medicine, Fukushima Medical University, 1 Hikarigaoka, Fukushima 960-1295, Japan; 4 Department of Public Health, School of Medicine, Fukushima Medical University, 1 Hikarigaoka, Fukushima 960-1295, Japan; 5 Fukushima Medical Center for Children and Women, Fukushima Medical University, 1 Hikarigaoka, Fukushima 960-1295, Japan

**Keywords:** dietary inflammatory index, preconception, oxidative stress, pregnancy, gestational diabetes mellitus, birth cohort study

## Abstract

We aimed to examine the impact of a preconception pro-inflammatory diet on gestational diabetes mellitus (GDM) using singleton pregnancy data from the Japan Environment and Children’s Study involving live births from 2011 to 2014. Individual meal patterns before pregnancy were used to calculate the dietary inflammatory index (DII). Participants were categorized according to DII quartiles 1–4 (Q1 and Q4 had the most pro-inflammatory and anti-inflammatory diets, respectively). The participants were stratified into five groups by pre-pregnancy body mass index (BMI): G1 to G5 (<18.5 kg/m^2^, 18.5 to <20.0 kg/m^2^, 20.0 to <23.0 kg/m^2^, 23.0 to <25.0 kg/m^2^, and ≥25.0 kg/m^2^, respectively). A multiple logistic regression model was used to estimate the effect of the anti-inflammatory diet on GDM, early diagnosed (Ed)-GDM, and late diagnosed (Ld)-GDM in each BMI group. Trend analysis showed that the risk of GDM, Ed-GDM, and Ld-GDM increased with increased pre-pregnancy BMI values. In the G4 group, the risk of Ed-GDM increased in Q2 and Q4. This study suggests that, although higher maternal BMI increases the risk of GDM, the effect of a preconception pro-inflammatory diet on the occurrence of GDM depends on pre-pregnancy BMI. This result may facilitate personalized preconception counseling based on maternal BMI.

## 1. Introduction

Gestational diabetes mellitus (GDM) is defined as a carbohydrate intolerance provoked by pregnancy [1]. In Japan, GDM occurs in approximately 1.8% of all cases [2], and it is a global public concern because of its long-term effects on the health of both mother and child. Approximately 70% of women with GDM develop diabetes mellitus (DM) within 22–28 years after pregnancy [3]. Offspring delivered by GDM women are at risk of poor neurodevelopmental outcomes, leading to obesity, impaired glucose tolerance, and DM in the future [4,5,6].

Pre-pregnancy body mass index (BMI) can be considered a proxy for health status because obesity not only results from an unhealthy dietary pattern, typically characterized by high caloric intake and low vitamin intake, but also from a sedentary lifestyle. There is strong evidence that maternal obesity increases the risk of GDM. Epidemiological studies have reported that the risk of GDM is four to eight times higher in overweight/obese women than in those with normal weight [7]. Recently, the importance of preconception care for obese women that involves altering their daily diet has grown as counseling or interventions to reduce the prevalence of GDM are being provided [8,9]. The dietary inflammatory index (DII) is a novel method for assessing personal daily diet to determine whether it is pro- or anti-inflammatory [10]. We have previously reported that a high DII score, which indicates a pro-inflammatory effect, a year before pregnancy, is associated with systemic inflammation [11].

Systemic inflammation and oxidative stress are closely related to pathophysiological processes, and one easily induces the other [12]. Oxidative stress plays a central role in the pathogenesis of several chronic diseases. Therefore, both processes occur simultaneously in many pathological conditions. One of the oxidative DNA damage products, 8-hydroxy-2′-deoxyguanosine (8-OHdG), is excreted directly in the urine and is a sensitive marker of oxidative stress [13]. Regarding the association between oxidative stress and GDM, oxidative stress could impair glucose tolerance and insulin resistance, and induce systemic endothelial dysfunction. These factors may directly or indirectly contribute to impaired pancreatic beta-cell function and glucose intolerance [14] and imply that a high DII score before pregnancy increases the risk of GDM via both maternal systemic inflammation and oxidative stress.

Few studies have been conducted to examine the association between a high DII and the occurrence of GDM [15,16], and the results suggest that a higher DII score, indicating a higher inflammatory effect, is associated with a risk of GDM. However, these studies have potential limitations: the setting was a developing country and a cross-sectional or prospective cohort study design with small sample sizes was used while simultaneously measuring maternal oxidative stress. Further evidence, especially from high-quality prospective study designs and carefully controlled trials, is required before providing solid recommendations regarding the association between the DII and the risk of GDM based on maternal BMI [17]. We hypothesized that a preconception high DII diet might increase the risk of GDM via maternal inflammation and oxidative stress. In this study, we aimed to investigate the effect of a pre-pregnancy pro-inflammatory diet based on the DII score on GDM using data from the largest Japanese birth cohort study. As the risk of GDM depends on pre-pregnancy BMI, we conducted this study by further stratifying pre-pregnancy BMI.

## 2. Material and Methods

### 2.1. The Japan Environment and Children’s Study (JECS)

We used data from the JECS, which is a government-funded longitudinal birth cohort study [18]. The JECS was started in January 2011 to investigate the effects of several environmental factors on the future health of children. This study was conducted at 15 regional centers across Japan, and the protocol has been reported elsewhere [18]. The eligibility criteria for the JECS participants were: (1) living in one of the study areas at the time of recruitment and expected to continually reside in Japan; (2) an expected delivery date between August 1, 2011, and mid-2014; and (3) no difficulty in writing and reading Japanese. The JECS protocol was reviewed and approved by the Ministry of the Environment’s Institutional Review Board on Epidemiological Studies and the ethics committees of all participating institutions. The JECS was conducted in accordance with the principles of the Declaration of Helsinki, and written informed consent was obtained from all participants.

### 2.2. Data Collection

We used the dataset released in March 2018 (jecs-an-20180131) for this study. This dataset consisted of three types of information: (1) self-reported medical background including pre-pregnancy BMI, maternal education, parity, method of conception, smoking status, and dietary pattern using a food frequency questionnaire (FFQ); (2) medical record transcripts of co-operating health care providers containing data on maternal background such as maternal age at delivery, presence of chronic hypertension before pregnancy, and obstetric outcomes, including timing of the diagnosis of GDM; and (3) maternal blood sample test results obtained during the first trimester. In the present analysis, we used data from the FFQ completed during the first trimester and collected information on the participants’ diet for one year until the current pregnancy, suggesting a preconception dietary pattern [11,19,20,21,22]. The FFQ was self-administered in the JECS and in other previous Japanese epidemiological studies [23].

### 2.3. Calculation of DII

The DII score is a comprehensive indicator of daily inflammatory and anti-inflammatory meal contents developed by Shivappa et al. [10]. The greater the DII score, the greater the diet’s pro-inflammatory effect. A higher negative value indicates a more anti-inflammatory diet. The method of calculating the DII in the JECS data has been previously reported [11,20,24]. Based on a previous study, the following 30 food parameters were obtained from each participant’s FFQ: energy; carbohydrate; protein; total fat; alcohol; fiber; cholesterol; saturated fat; monounsaturated fatty acids; polyunsaturated FAs; FAs (n–3 and n–6 FAs); niacin; thiamine; riboflavin; iron; magnesium; zinc; selenium; folic acid; β-carotene; vitamin A, B-12, B-6, C, D, and E; garlic; ginger; and onion [23]. The DII score of each participant was calculated as follows: first, dietary data were linked to a worldwide database that provided a robust estimate of the mean and standard deviation (SD) for each parameter included in the DII [16]. The Z-score was calculated by subtracting the standard global mean from the reported amount and dividing the result by the SD. The Z scores were not normally distributed (right skewing); thus, the Z-score for each value was converted to a centered percentile score. Then, the centered percentile score for each food parameter was multiplied by the respective food parameter effect score (obtained by reviewing a total of 1943 research articles to determine the relationship between food parameters and inflammation, as well as by scoring) to obtain a food parameter-specific DII score, which was summed to create the overall DII score for each participant. DII = I1∙P1 + I2∙P2 + … + I30∙P30, where I is the food parameter effect score considering the effect of inflammation obtained from reviewed research articles, and P is the food-specific centered percentile score derived from food data. The minimum/maximum DII scores in pregnant populations in a previous JECS study were reported to range from −6.16 to +5.80 [11].

### 2.4. Measurement of 8-OHdG Levels

Urine 8-OHdG levels (ng/mL) were estimated during the second and third trimesters using liquid chromatography-tandem mass spectrometry. Urinary creatinine level was determined as the proportion of 8-OHdG excreted in the urine [25].

### 2.5. Obstetric Outcomes and Confounding Factors

All pregnant women participating in the JECS underwent a screening procedure for GDM in both early and late pregnancies. In Japan, glucose tolerance screening and testing for GDM is performed for every pregnant woman, according to the protocols recommended by the Obstetrics Society and Diabetes Society of Japan. Depending on the local obstetrics institution, it is a two-step protocol during both the first and second/third trimesters [19,26,27]. Briefly, the first step is the screening of random blood glucose (RBG) levels or fasting 1-h 50-g oral glucose challenge test (GCT) during the first trimester. If the screening was positive, the pregnant women underwent a 75-g oral glucose tolerance test (OGTT) and are confirmed as having GDM. If the first trimester screening was negative, the women underwent a second screening using either RBG or a fasting 1-h 50-g GCT in the second/third trimester. An RBG level ≥ 95 mg/dL or a GCT level > 140 mg/dL was considered a positive screening result. In the case of a positive screening result, a 75-g OGTT was conducted with cutoff values of ≥92 mg/dL for fasting plasma glucose, ≥180 mg/dL for plasma glucose at 1 h, and ≥153 mg/dL for plasma glucose at 2 h. GDM was confirmed if at least one of the three aforementioned glycemic levels was above the recommended threshold during the OGTT (fasting plasma glucose, plasma glucose at 1 h, and plasma glucose at 2 h). As Japan has a unique GDM screening system conducted during two pregnancy periods (early and mid-trimesters), we further categorized GDM into early-onset (Ed) GDM (diagnosed before 24 weeks) and late-onset (Ld) GDM (diagnosed after 24 weeks) [19,27]. Therefore, a case of GDM consisted of both “Ed-GDM” and “Ld-GDM” at preset analysis. To elucidate the onset of glucose intolerance during pregnancy, participants with DM before pregnancy, maternal serum glycated hemoglobin levels ≥ 6.5% in the first trimester, and those who used any steroid during pregnancy were excluded from the present study.

The following items were considered confounding: maternal age at the time of delivery, method of conception, maternal smoking status, maternal educational status, and chronic hypertension before pregnancy. Maternal age at delivery was categorized into four groups: <20 years, 20–29 years, 30–39 years, and ≥40 years. In the JECS study, the method of conception was categorized as assisted reproductive technology (ART) or non-ART. ART pregnancy in this data set was defined as conception after in vitro fertilization and intracytoplasmic sperm injection or as cryopreserved, frozen, or blastocyst embryo transfers [28]. A self-report questionnaire during the first trimester had the following options regarding smoking history: “Never,” “Previously did, but quit before recognizing current pregnancy,” “Previously did, but quit after finding out current pregnancy,” and “Yes, I still smoke”. While women who chose “Currently smoking” were considered smokers (smoking), the others were considered non-smokers (non-smoking). Based on the Japanese educational system, maternal education is categorized into the following four groups: junior high school, <10 years; high school, 10–12 years; professional school or university, 13–16 years; and graduate school, ≥17 years of education [2]. Maternal chronic hypertension was defined as the presence of hypertension (systolic blood pressure >140 mm Hg or diastolic blood pressure >90 mm Hg) before conception. The mothers were also categorized as primipara or multipara, based on the number of previous deliveries.

BMI was calculated according to World Health Organization standards (body weight (kg) / height^2^ (m^2^)). The participants were categorized into five groups according to their BMI before pregnancy: group 1 (G1), <18.5 kg/m^2^; group 2 (G2), 18.5 to <20.0 kg/m^2^; group 3 (G3), 20.0 to <23.0 kg/m^2^; group 4 (G4), 23.0 to <25.0 kg/m^2^; and group 5 (G5), >25.0 kg/m^2^ [29,30,31].

### 2.6. Statistical Analysis

Participants were categorized into quartiles 1–4 (Q1 had the most pro-inflammatory diet, whereas Q4 had the most anti-inflammatory diet) and further stratified into five groups based on pre-pregnancy BMI. Maternal characteristics, obstetric outcomes, and median urine 8-OHdG levels were summarized according to the DII and pre-pregnancy BMI categories. A one-way analysis of variance and chi-square test were used to compare continuous and categorical variables, respectively. To compare the median urinary 8-OHdG levels, the Kruskal–Wallis analysis was conducted. The extended Mantel–Haenszel chi-square test for linear trends was used to analyze the trends in obstetric outcomes among both the DII and pre-pregnancy BMI categories. The adjusted odds ratios (aOR) and 95% confidence intervals (CIs) for GDM, Ed-GDM, and Ld-GDM were calculated using multiple logistic regression modeling. Age, maternal education, smoking status, ART pregnancy, maternal chronic hypertension at the time of pregnancy, and parity were used to calculate aOR. A dummy variable was used for categorical variables with more than three categories. All statistical analyses were performed using SPSS version 26 (IBM Corp., Armonk, NY, USA). Statistical significance was set at *p* < 0.05.

## 3. Results

**Figure 1:**
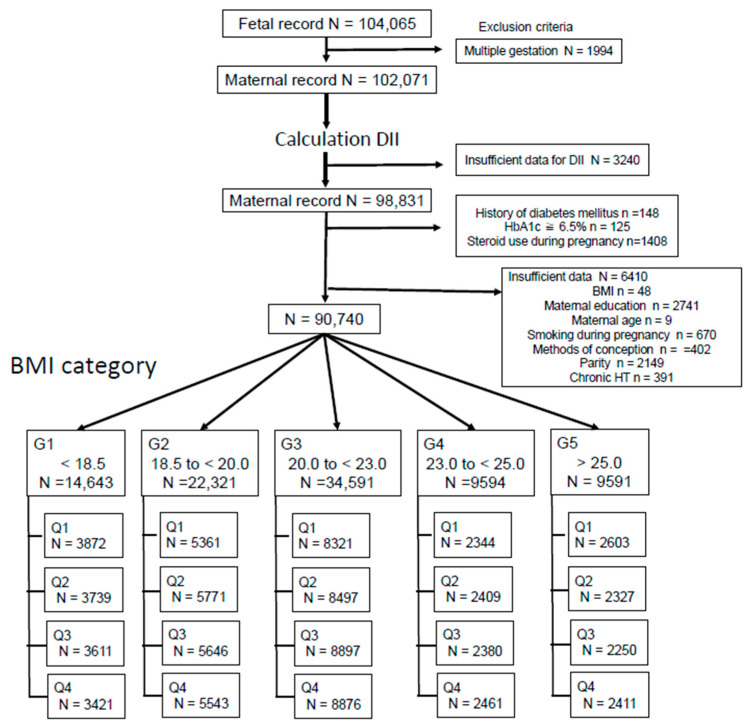
Study flow.

Figure 1 shows the participant selection process. After applying the exclusion criteria, 90,740 mothers were included in the present analysis: 2405 (2.7%) GDM cases, 705 (0.8%) Ed-GDM cases, and 1447 (1.8%) Ld-GDM cases. In 153 GDM cases, the gestational age at the time of GDM diagnosis was unknown. The mothers were further divided into five groups: G1: BMI <18.5 kg/m^2^ (*n* = 14,643), G2: BMI: 18.5 to <20.0 kg/m^2^ (*n* = 22,321), G3: BMI: 20.0 to <23.0 kg/m^2^ (*n* = 34,591), G4: BMI: 23.0 to <25.0 kg/m^2^ (*n* = 9594), and G5: BMI >25.0 kg/m^2^ (*n* = 9591). Participants in each group were further categorized into four groups based on the DII (Q1: the group with the most pro-inflammatory diet and Q4: the group with the highest anti-inflammatory diet) (Figure 1).

Table 1 shows the maternal background and occurrence of GDM based on the DII category. The proportions of participants in the 30 to 39 years and ≥40 years groups were highest in the Q4 group (both *p* < 0.001). The proportions of those with BMI <18.5 kg/m^2^ and ≥25.0 kg/m^2^ were highest in Q1 (*p* < 0.001). The proportions of primiparas, current smokers, those with <10 years of education, and those with a white blood cell count >9000 /μL were highest in Q1 (all *p* < 0.001). The mean white blood cell count and median urine 8-OHdG level were significantly different among the four quartiles and highest in Q1 (*p* < 0.001 for both). There was no significant difference in the rate of chronic hypertension during pregnancy (*p* = 0.443) and the occurrence of GDM (*p* = 0.256), Ed-GDM (*p* = 0.073), and Ld-GDM (*p* = 0.916) among the five BMI groups. 

Table 2 shows the maternal background and occurrence of GDM based on pre-pregnancy BMI. The mean maternal age, white blood cell count, proportion of smokers, rate of chronic hypertension, and proportion of those with a white blood cell count >9000 /μL were highest in the G5 group (BMI > 25.0; all *p* < 0.001). The median urine 8-OHdG level was significantly different among the five groups and highest in the G5 group (*p* < 0.001). The mean DII score was significantly different among the five BMI groups and highest in the G1 group (BMI < 18.5 kg/m^2^). The extended Mantel–Haenszel chi-square test for linear trends showed that the occurrence of GDM, Ed-GDM, and Ld-GDM increased with BMI category increase (all *p* < 0.001). 

Table 3 shows the relationship between the DII and the risk of developing GDM. In the G4 category (BMI 23.0 to <25.0 kg/m^2^), when Q1 (the most pro-inflammatory diet) was set as the reference, GDM risk increased in Q2 (aOR: 1.74, 95% CI: 1.20–2.50), Q3 (aOR: 1.59, 95% CI: 1.09–2.30), and Q4 (aOR: 1.75, 95% CI: 1.21–2.52).

Table 4 shows the relationship between the DII and the risk of Ed-GDM. In the G4 category (BMI 23.0 to <25.0 kg/m^2^), Ed-GDM risk increased in Q2 (aOR: 2.41, 95% CI: 1.16–4.99) and Q4 (aOR: 2.11, 95% CI: 1.02–4.40). In the G5 category (BMI > 25.0 kg/m^2^), Ed-GDM risk increased in Q2 (aOR: 1.41, 95% CI: 1.00–1.98).

Table 5 shows the relationship between the DII and the risk of Ld-GDM. No significant relationship was observed in any of the BMI categories.

## 4. Discussion

In this study, we found that (1) consumption of a high DII diet before pregnancy increased both maternal systemic inflammation during early pregnancy and maternal oxidative stress during the second trimester. (2) Although the risk of GDM increased along with the pre-pregnancy BMI category, consuming a high DII diet before pregnancy did not increase BMI during pregnancy, as Table 2 shows that G1, which indicates the lowest BMI category, had the highest mean DII score. (3) Women with a BMI ranging from 23.0 to <25.0 kg/m^2^ during pregnancy had an increased risk of GDM, despite consuming an anti-inflammatory diet before pregnancy.

Several studies have focused on the correlation between the DII and non-communicable diseases, such as cancer, cardiovascular disease, obesity, respiratory disease, and mental health disorders [17]. To date, only a few previous studies have specifically assessed the association between the consumption of a pro-inflammatory diet and the incidence of GDM. A previous case-control study conducted among Iranian women with 122 cases and 266 controls found that the consumption of a pro-inflammatory diet was associated with increased odds of developing GDM (OR: 2.10, 95% CI: 1.02, 4.34) [16]. In a Chinese cohort study, Zhang et al. reported that a higher DII score was associated with a higher risk of GDM, particularly among women who were overweight or obese before pregnancy [15]. Using the same data set in JECS and calculating each participant’s DII, we have previously reported that a high DII score (indicating a pro-inflammatory diet) is associated with preterm birth (PTB), low birth weight (LBW), hypertension disorders of pregnancy, and fetal hypoxia among primiparas, and a preconception low DII (indicating anti-inflammatory diet) decreases the risk of PTB and LBW, especially among women with endometriosis [11,20,24]. Japan has a unique and universal screening procedure for GDM diagnosis. As a result, GDM cases are identified as Ed-GDM and Ld-GDM [19,27]. We previously reported that Ed-GDM, which is diagnosed before 24 gestational weeks, was associated with adverse obstetric complications, such as early- and late-onset hypertensive disorders of pregnancy. We expected that a high DII would increase the risk of some phenotypes (Ed or Ld) of GDM because the high DII in this study is associated with leukocytosis and oxidative stress, which impairs glucose tolerance and insulin resistance, and induces systemic endothelial dysfunction, resulting in direct or indirect contribution to impaired pancreatic beta-cell function and glucose intolerance [19]. Unlike many previous reports, consuming an anti-inflammatory diet before pregnancy leads to a higher likelihood of developing GDM and Ed-GDM among women with a BMI ranging from 23.0 to <25.0 kg/m^2^ in this study. The reason for the counterintuitive correlation between a low DII score before pregnancy and increased risk of GDM is speculative. Sen et al. found that the consumption of a high DII diet during pregnancy was associated with a lower likelihood of GDM diagnosis, particularly among women with a BMI ranging from 25.0 to <30.0 kg/m^2^ [32]. Radesky et al. found that higher animal fat and cholesterol intake were associated with the development of GDM, but there was no association between carbohydrate intake and the development of GDM [33]. In overweight women, who are more likely to develop GDM, a higher intake of carbohydrates could have contributed to this unexpected correlation between the DII and GDM [32].

Overweight and obesity are often the consequences of an accumulation of unhealthy lifestyles. Recently, interest in preconception health has grown as preconception is a crucial period for influencing not only pregnancy outcomes but also the long-term health of the mother and child [34]. Therefore, lifestyle interventions for women before pregnancy may capitalize on a “window period” when women appear to be more motivated to engage in behavioral changes [20,21]. There is no doubt that high BMI leads to an increase in future maternal and neonatal risks, and that the consumption of a pro-inflammatory diet has adverse effects on long-term maternal health [11,20,24]. However, this study showed that the lowest BMI group had the highest mean DII score before pregnancy. Therefore, personalized preconception counseling to alter nutritional habits and encourage appropriate weight gain during pregnancy can become a potential healthcare management strategy to promote long-term health in mothers and their offspring, based on maternal pre-pregnancy BMI. In this study, we found an unexpected result showing that a high DII score before pregnancy could lead to a low BMI. In the future, we will investigate the difference in metabolic mechanisms between the consumption of a high DII diet before pregnancy, resulting in low or high BMI during pregnancy. 

The strength of this study is the large sample size and large-scale data obtained from the JECS supported by the Japanese government. The participants represented the general pregnant population in Japan [18]. Although the JECS is not a randomized controlled study, the large-scale nature of this cohort study enables the evaluation of associations between pre-pregnancy nutritional status and obstetric outcomes. This study had potential limitations. First, the data did not include glycemic conditions, such as the results of the RBG test, fasting GCT, and OGTT, which may have affected the obstetric outcomes [35]. Second, we are not aware of any medical interventions in the GDM cases, which might also have affected the obstetric outcomes [36]. Third, since the FFQ in the JECS targeted Japanese women and focused on Japanese food customs, the findings may not apply to other ethnicities [20].

This study indicated that although the DII score before pregnancy affected the risk of GDM, these risks depended on maternal pre-pregnancy BMI. Few studies have comprehensively analyzed preconception diet patterns, maternal oxidative stress, maternal inflammation, and BMI before pregnancy to estimate the risk of GDM. We hope that the present study will form the basis for appropriate personalized counseling as a form of preconception care to reduce the risk of GDM based on BMI to improve future maternal and neonatal health.

## Figures and Tables

**Table 1:** Maternal medical background and obstetric outcomes based on the dietary inflammatory index (DII).

Variable	Q1 (Most Proinflammatory Group)	Q2	Q3	Q4 (Most Anti-Inflammatory Group)	*p*-Value
	*n* = 22,501	*n* = 22,743	*n* = 22,784	*n* = 22,712	
Maternal background					
DII, mean (SD)	3.41 (0.91)	0.99 (0.60)	−1.03 (0.59)	−3.40 (0.90)	<0.001 ^a^
Maternal age, mean year (SD)	29.8 (5.2)	31.1 (4.8)	31.7 (4.9)	32.1 (4.8)	<0.001 ^a^
Maternal age category, %					
≤19	1.6	0.6	0.6	0.4	<0.001 ^b^
20–29	47.2	37.3	32.4	29.2	
30–39	48.0	57.8	61.9	64.6	
≥40	3.2	4.3	5.1	5.8	
BMI, mean (SD)	21.3 (3.4)	21.2 (3.2)	21.2 (3.2)	21.3 (3.3)	<0.001 ^a^
BMI, %					
<18.5	17.2	16.4	15.8	15.1	<0.001 ^b^
18.5–19.9	23.8	25.4	24.8	24.4	
20.0–22.9	37.0	37.4	39.0	39.1	
23.0–24.9	10.4	10.6	10.4	10.8	
≥25.0	11.6	10.2	9.9	10.6	
Primipara, %	48.8	41.5	38.0	33.1	<0.001 ^b^
Smoking, %	6.8	4.7	3.9	3.8	<0.001 ^b^
ART, %	2.2	2.9	3.2	3.4	<0.001 ^b^
Chronic HT, %	1.3	1.2	1.1	1.1	0.443
Maternal education (years), %					
<10	7.3	4.6	3.8	3.5	<0.001 ^b^
10–12	39.5	31.9	28.4	26.6	
13–16	38.1	42.1	43.4	44.7	
≥17	15.2	21.4	24.5	25.2	
WBC, counts per liter, mean (SD)	8081 (1948)	8045 (1934)	8011 (1933)	8003 (1925)	<0.001 ^a^
WBC >9000 (counts per liter), %	26.9	26.0	25.3	25.0	<0.001 ^b^
Urine 8-OHdG levels, median (IQR)	1.93 (1.17–2.84)	1.84 (1.10–2.73)	1.77 (1.04–2.64)	1.70 (0.99–2.59)	<0.001 ^c^
Obstetrics outcomes					
GDM, %	2.5	2.7	2.7	2.7	0.256 ^b^
Ed-GDM, %	0.7	0.8	0.8	0.9	0.073 ^b^
Ld-GDM, %	1.6	1.6	1.6	1.6	0.961 ^b^

SD: standard deviation, BMI: body mass index, ART: assisted reproductive technology, HT: hypertension, WBC: white blood cell, 8-OHdG: 8-hydroxy-2′-deoxyguanosine, IQR: interquartile range, GDM: gestational diabetes mellitus, Ed: early diagnosed, Ld: late diagnosed. ^a^ *p*-value, one-way analysis of variance; ^b^ *p*-value, chi-squared test; ^c^ *p*-value, Kruskal–Wallis analysis.

**Table 2:** Maternal background and obstetrics outcomes according to body mass index (BMI) before pregnancy.

BMI Category	<18.5 (G1)	18.5 to <20.0 (G2)	20 to <23.0 (G3)	23.0 to <25.0 (G4)	>25 (G5)	*p*-Value
Number of patients	14,643	22,321	34,591	9594	9591	
BMI, mean (SD)	17.6 (0.7)	19.3 (0.4)	21.3 (0.8)	23.9 (0.6)	29.4 (3.3)	<0.001 ^a^
Maternal age, mean year (SD)	30.3 (5.0)	31.0 (4.9)	31.4 (5.0)	31.8 (5.1)	31.8 (5.1)	<0.001 ^a^
Smoking, %	5.5	4.1	4.3	5.0	6.9	<0.001 ^b^
Chronic HT, %	0.5	0.6	0.9	1.4	4.5	<0.001 ^c^
DII, median (IQR)	0.14 (−1.91–2.18)	−0.03 (−2.05–1.97)	−0.12 (−2.12–1.97)	−0.05 (−2.12–2.00)	0.10 (−2.07–2.24)	<0.001 ^c^
Urine 8-OHdG levels, ng/mL, median (IQR)	1.81 (1.07–2.71)	1.78 (1.04–2.66)	1.77 (1.04–2.65)	1.84 (1.11–2.71)	2.02 (1.24–2.98)	<0.001 ^c^
WBC, counts per liter, mean (SD)	7860 (1904)	7913 (1898)	7997 (1915)	8229 (1949)	8534 (2030)	<0.001 ^a^
WBC >9000 (counts per liter), %	22.7	23.3	25.1	29.4	35.1	<0.001 ^c^
GDM, %	1.6	1.6	2.2	3.2	7.8	<0.001 ^d^
Ed-GDM, %	0.3	0.5	0.5	0.9	2.8	<0.001 ^d^
Ld-GDM, %	1.0	0.9	1.4	1.9	4.2	<0.001 ^d^

SD: standard deviation, HT: hypertension, DII: dietary inflammatory index, IQR: interquartile range, 8-OHdG: 8-hydroxy-2′-deoxyguanosine, WBC: white blood cell, GDM: gestational diabetes mellitus, Ed: early diagnosed, Ld: late diagnosed. ^a^ *p*-value, one-way analysis of variance; ^b^ *p*-value, chi-squared test; ^c^ *p*-value, Kruskal–Wallis analysis; ^d^ *p*-value, extended Mantel–Haenszel chi-squared test for linear trends.

**Table 3:** Relationship between the dietary inflammatory index DII and the risk for gestational diabetes mellitus (GDM).

BMI Category		Q1 (Most Proinflammatory Group)	Q2	Q3	Q4 (Most Anti-Inflammatory Group)
G1					
	Number	3872	3739	3611	3421
	Case, %	1.5	1.6	1.7	1.5
	OR (95% CI)	Ref	1.04 (0.72–1.49)	1.09 (0.76–1.56)	0.98 (0.67–1.43)
	aOR (95% CI)	Ref	0.97 (0.67–1.39)	0.99 (0.69–1.43)	0.88 (0.60–1.29)
G2					
	Number	5361	5771	5646	5543
	Case, %	1.7	1.4	1.7	1.8
	OR (95% CI)	Ref	0.82 (0.61–1.12)	1.00 (0.75–1.34)	1.07 (0.80–1.42)
	aOR (95% CI)	Ref	0.73 (0.54–1.00)	0.86 (0.64–1.16)	0.88 (0.66–1.19)
G3					
	Number	8321	8497	8897	8876
	Case, %	2.0	2.3	2.3	2.1
	OR (95% CI)	Ref	1.16 (0.94–1.43)	1.14 (0.93–1.40)	1.06 (0.86–1.31)
	aOR (95% CI)	Ref	1.07 (0.87–1.32)	1.02 (0.83–1.26)	0.93 (0.75–1.15)
G4					
	Number	2344	2409	2380	2461
	Case, %	2.0	3.6	3.4	3.8
	OR (95% CI)	Ref	1.85 (1.29–2.66)	1.76 (1.22–2.54)	1.96 (1.37–2.81)
	aOR (95% CI)	Ref	1.74 (1.20–2.50)	1.59 (1.09–2.30)	1.75 (1.21–2.52)
G5					
	Number	2603	2327	2250	2411
	Case, %	7.4	8.4	7.4	7.9
	OR (95% CI)	Ref	1.14 (0.93–1.41)	1.00 (0.81–1.24)	1.07 (0.87–1.32)
	aOR (95% CI)	Ref	1.12 (0.91–1.39)	0.96 (0.77–1.19)	1.02 (0.82–1.26)

BMI: body mass index, OR: odds ratio, aOR: adjusted odds ratio, CI: confidence interval, Ref: reference. The aOR was calculated by logistic regression analysis using maternal age, maternal education, maternal smoking status, whether or not assisted reproductive technology was used, presence/absence of maternal chronic hypertension at the time of pregnancy, and parity.

**Table 4:** Relationship between the dietary inflammatory index and the risk of early diagnosed gestational diabetes mellitus (Ed-GDM).

BMI Category		Q1 (Most Proinflammatory Group)	Q2	Q3	Q4 (Most Anti-Inflammatory Group)
G1					
	Number	3872	3739	3611	3421
	Case, %	0.3	0.3	0.4	0.4
	OR (95% CI)	Ref	1.04 (0.45–2.39)	1.37 (0.62–3.01)	1.44 (0.65–3.18)
	aOR (95% CI)	Ref	0.99 (0.42–2.31)	1.27 (0.57–2.84)	1.33 (0.59–2.98)
G2					
	Number	5361	5771	5646	5543
	Case, %	0.6	0.4	0.5	0.6
	OR (95% CI)	Ref	0.65 (0.37–1.14)	0.98 (0.59–1.62)	1.00 (0.60–1.65)
	aOR (95% CI)	Ref	0.55 (0.32–0.97)	0.79 (0.47–1.31)	0.76 (0.46–1.27)
G3					
	Number	8321	8497	8897	8876
	Case, %	0.4	0.5	0.6	0.6
	OR (95% CI)	Ref	1.27 (0.81–1.99)	1.49 (0.97–2.29)	1.41 (0.91–2.18)
	aOR (95% CI)	Ref	1.15 (0.73–1.80)	1.29 (0.84–2.00)	1.19 (0.76–1.85)
G4					
	Number	2344	2409	2380	2461
	Case, %	0.4	1.2	0.8	1.1
	OR (95% CI)	Ref	2.75 (1.33–5.66)	1.98 (0.92–4.24)	2.69 (1.30–5.54)
	aOR (95% CI)	Ref	2.41 (1.16–4.99)	1.61 (0.74–3.47)	2.11 (1.02–4.40)
G5					
	Number	2603	2327	2250	2411
	Case, %	2.4	3.4	2.6	3.1
	OR (95% CI)	Ref	1.40 (1.00–1.96)	1.07 (0.74–1.53)	1.28 (0.91–1.80)
	aOR (95% CI)	Ref	1.41 (1.00–1.98)	1.06 (0.73–1.53)	1.26 (0.89–1.78)

BMI: body mass index, OR: odds ratio, aOR: adjusted odds ratio, CI: confidence interval, Ref: reference. The aOR was calculated by logistic regression analysis using maternal age, maternal education, maternal smoking status, whether or not assisted reproductive technology was used, presence/absence of maternal chronic hypertension at the time of pregnancy, and parity.

**Table 5:** Relationship between the dietary inflammatory index and the risk of late diagnosis gestational diabetes mellitus (Ld-GDM).

BMI Category		Q1 (Most Proinflammatory Group)	Q2	Q3	Q4 (Most Anti-Inflammatory Group)
G1					
	Number	3872	3739	3611	3421
	Case, %	1.1	1.1	1.0	1.0
	OR (95% CI)	Ref	1.06 (0.69–1.64)	0.97 (0.62–1.51)	0.91 (0.57–1.44)
	aOR (95% CI)	Ref	1.01 (0.65–1.57)	0.92 (0.58–1.44)	0.86 (0.54–1.37)
G2					
	Number	5361	5771	5646	5543
	Case, %	1.0	0.8	0.9	1.1
	OR (95% CI)	Ref	0.89 (0.60–1.32)	0.95 (0.64–1.40)	1.12 (0.77–1.63)
	aOR (95% CI)	Ref	0.84 (0.56–1.24)	0.88 (0.59–1.30)	1.02 (0.69–1.50)
G3					
	Number	8321	8497	8897	8876
	Case, %	1.4	1.6	1.4	1.3
	OR (95% CI)	Ref	1.13 (0.88–1.45)	1.02 (0.80–1.32)	0.94 (0.72–1.21)
	aOR (95% CI)	Ref	1.05 (0.82–1.35)	0.92 (0.72–1.19)	0.83 (0.63–1.07)
G4					
	Number	2344	2409	2380	2461
	Case, %	1.5	1.9	2.1	2.2
	OR (95% CI)	Ref	1.26 (0.80–1.96)	1.42 (0.92–2.19)	1.51 (0.98–2.31)
	aOR (95% CI)	Ref	1.22 (0.78–1.91)	1.34 (0.86–2.09)	1.43 (0.93–2.22)
G5					
	Number	2603	2327	2250	2411
	Case, %	4.3	4.2	4.0	4.2
	OR (95% CI)	Ref	0.97 (0.74–1.28)	0.92 (0.69–1.22)	0.96 (0.73–1.27)
	aOR (95% CI)	Ref	0.94 (0.71–1.24)	0.86 (0.65–1.15)	0.89 (0.67–1.17)

BMI: body mass index, OR: odds ratio, aOR: adjusted odds ratio, CI: confidence interval, Ref: reference. The aOR was calculated by logistic regression analysis using maternal age, maternal education, maternal smoking status, whether or not assisted reproductive technology was used, presence/absence of maternal chronic hypertension at the time of pregnancy, and parity.

## Data Availability

Data are unsuitable for public deposition due to ethical restrictions and legal framework of Japan. It is prohibited by the Act on the Protection of Personal Information (Act No.57 of 30 May 2003, amendment on 9 September 2015) to publicly deposit the data containing personal information. Ethical Guidelines for Epidemiological Research enforced by the Japan Ministry of Education, Culture, Sports, Science and Technology and the Ministry of Health, Labour and Welfare also restricts the open sharing of the epidemiologic data. All inquiries about access to data should be sent to: jecs-en@nies.go.jp. The person responsible for handling enquiries sent to this e-mail address is Dr Shoji F. Nakayama, JECS Programme Office, National Institute for Environmental Studies.
